# Popliteal Arteriovenous Fistula Diagnosed Eight Years after Total Knee Arthroplasty. Endovascular Treatment with Viabahn^®^ Endoprosthesis and Five-Year Follow-Up

**DOI:** 10.3390/reports7030059

**Published:** 2024-07-25

**Authors:** Francisco Santiago Lozano-Sánchez, Jesús García-Alonso, Roberto Salvador-Calvo, Luis Velasco-Pelayo, María Begoña García-Cenador

**Affiliations:** 1 Angiology and Vascular Surgery Service, Salamanca University Hospital (CAUSA), 37008 Salamanca, Spain; lozano@usal.es (F.S.L.-S.); robersc81@hotmail.com (R.S.-C.); 2 Institute of Biomedical Research of Salamanca (IBSAL), 37007 Salamanca, Spain; jgarciaal@saludcastillayleon.es (J.G.-A.); lvelascop@saludcastillayleon.es (L.V.-P.); 3 Department of Surgery, University of Salamanca (USAL), 37007 Salamanca, Spain; 4 Radiodiagnostic Service, Salamanca University Hospital (CAUSA), 37008 Salamanca, Spain

**Keywords:** popliteal artery injury, arteriovenous fistula, total knee arthroplasty, endovascular treatment

## Abstract

Background: Orthopedic surgery, while it rarely cause iatrogenic vascular lesions, leads to significant clinical, social, and economic consequences when it does. The knee is particularly susceptible to these injuries. Case Description: This case study presents the clinical case of a 71-year-old woman with a history of left total knee replacement. Eight years after the initial procedure, a popliteal—popliteal arteriovenous fistula was identified in the same knee. Given the location and caliber of the fistula, and despite the absence of symptoms, an endovascular prosthesis (Viabahn^®^) was deployed in the popliteal artery to cover the fistula. The prosthesis remained intact for the remainder of the patient’s life, who succumbed to metastatic cancer five years later. Additionally, a review of the literature was conducted. Conclusion: This brief report describes an exceptional case of popliteal arteriovenous fistula, diagnosed eight years after a TKA, treated endovascularly and followed up over five years. Both pseudoaneurysms and arteriovenous fistulae should also be considered for early detection.

## 1. Introduction

Orthopedic surgery infrequently leads to iatrogenic vascular lesions, which have profound clinical, social, and economic impacts. The knee is the part of the anatomy that is most prone to damage [1,2].

At the beginning of this century, a literature review reported a rate of vascular complications of 0.03–0.2% following total knee arthroplasty (TKA) [3]. The same review also reported 40 acute limb ischemia, 10 pseudoaneurysms, 9 direct lesions, and 1 arteriovenous fistula [3]. 

More extensive and more recent data provide greater precision. Thus, out of 1,419,557 TKAs, there have been 54 cases of major vascular lesions per 100,000 procedures (0.05%), with 21% amputations or permanent neurological deficits [4]. A systematic review (and meta-analysis) reports 66 major vascular lesions following TKA between 1998 and 2018, where the popliteal artery is the one most affected (58 cases), with the most frequent kind of injury being a pseudoaneurysm (26 cases), followed by arterial thrombosis (19 cases), arterial section (eight cases) and arteriovenous fistula (five cases) [4].

The literature reveals that the average time between a TKA and the diagnosis of an iatrogenic vascular lesion ranges from immediately/hours for a hemorrhage, days for arterial thrombosis, and weeks/months for pseudoaneurysms and arteriovenous fistulas [5].

This research describes an exceptional case of popliteal arteriovenous fistula, diagnosed eight years after a TKA, treated endovascularly and followed up over five years.

## 2. Case Description

On 13 March 2013, a 71-year-old woman presented with edema in her right leg. Her medical record involved arterial hypertension, type 2 diabetes mellitus, and hypothyroidism. She had three normal births and one miscarriage, no toxic habits and no known allergies.

In 2005, TKA of the right leg. In 2006, pertrochanteric fracture of the right leg (gamma nail). In 2009, breast cancer (tumorectomy) with pulmonary metastasis (lobectomy). Subsequent radiotherapy.

The patient had been attending the doctor for a period of eight years, during which time she had undergone a number of check-ups in relation to her knee prosthesis. It was not until she noticed a “throbbing” sensation in her right knee that she sought further medical attention. This was identified by the doctor as a thrill, at which point the diagnosis and treatment of the fistula were carried out.

At the level of the right knee, a thrill (fremitus) is palpated and a systolic reinforcement murmur is heard. Normal popliteal pulse and weak pedial and posterior tibial pulses. Ankle-Brachial Index (ABI) = 0.70.

**Figure 1:**
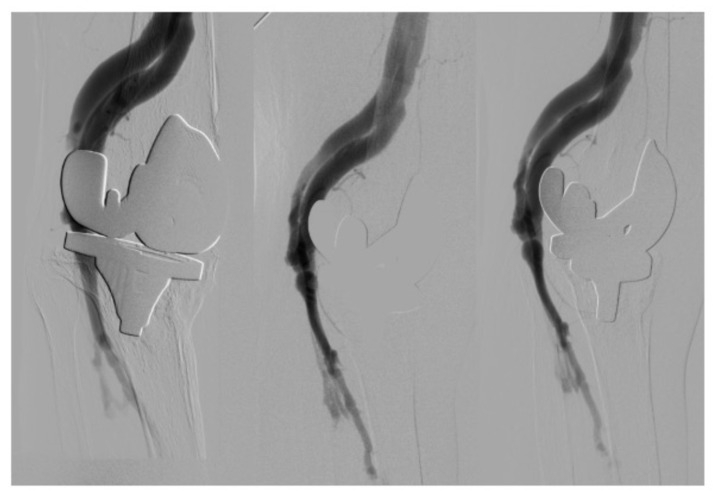
Diagnostic arteriography. Early contrast packing of the surface femoral and popliteal veins.

A physical examination finds a thrill (fremitus) and a murmur in the right knee. Palpable popliteal pulse, weak distal pulses. Iliotibial band (ITB) = 0.70. Normal lower left leg. The eco-Doppler reveals an arteriovenous fistula. An arteriography (Figure 1) confirms the presence of popliteal—popliteal arteriovenous fistula in the second portion, with a high flow.

**Figure 2:**
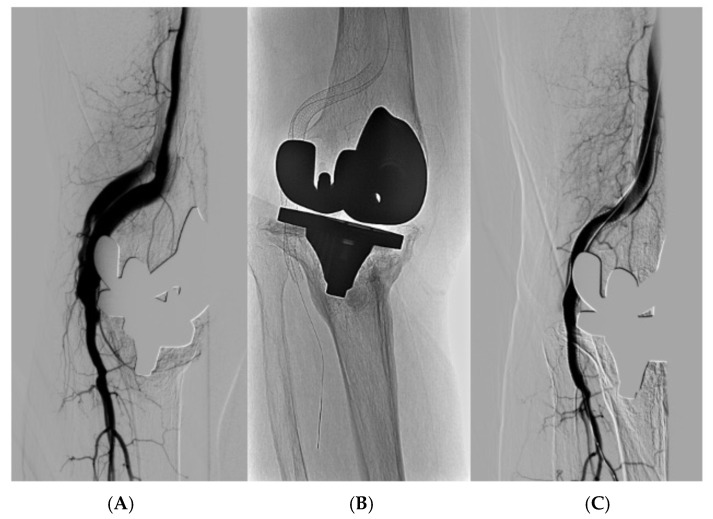
Therapeutic arteriography: (**A**) check-up after inserting Viabahn^®^ showing minimum packing of the vein sector; (**B**) Viabahn^®^ at the level of popliteal artery; (**C**) final check-up after intra-stent angioplasty (absence of contrast passing to the vein sector).

Informed of the therapeutic options (open and endovascular surgery), the patient chose an endovascular treatment, which was performed on 3 May 2013, with the successful insertion of a Viabahn^®^ (WL Gore and Associates) (Figure 2).

The patient was released from hospital after four days, with no complications. In the first check-up (13 June 2013), there is no palpable thrill and no audible murmur; ITB, ABI, 0.93 > 1. In subsequent check-ups, the endoprosthesis remains permeable. The last check-up is held on 5 April 2018 (five years after the endoprosthesis), when the patient records pulmonary, bone and hepatic metastases.

## 3. Discussion

Traumatic arteriovenous fistulas are a rare complication of TKA, occurring less frequently than other injuries such as arterial lacerations, thrombosis, and pseudoaneurysms [3,4]. We have found only five cases [5,6,7].

The average age of these patients was 75 (ranging from 62 to 79), involving three women and two men. They were all diagnosed after the first 24 h of the postop period, with one patient even being diagnosed three years after the TKA for presenting a persistent oedema (similar to our case) [7]. The clinical situation in all of them was non-specific: pain (two cases), pulsatile mass (one case), weak pulse (one case), and oedema (one case).

Ultrasounds were useful only in one case, while the remaining four cases required arteriography. They were all treated. Three patients initially received endovascular treatment. In the end, three required an open surgery procedure, and one patient underwent an amputation, with an average follow-up time of seven months (ranging from 3 to 12 months).

Iatrogenic arteriovenous fistulas in the popliteal region have also been reported as a complication of a knee arthroscopy. A review (1985-2014) describes 62 vascular lesions following a knee arthroscopy, mainly after meniscectomies, where isolated arteriovenous fistulas (three cases) or those associated with pseudoaneurysm (eight cases) were also the rarest type of injury [8]. Out of the 11 cases, only 3 received endovascular treatment, with a highlights being the cases of the Spanish surgeon and radiologist, respectively, Taboada and Capel (2009) [9], who used a similar prosthesis to the one used in our study. They clearly explain the limitations of stents in this area of flexion (compression, bending, migration, breakage, intimal hyperplasia, thrombosis, etc.); nonetheless, they highlight the properties of the self-expanding endoprosthesis Viabahn^®^, which is more flexible and resistant to bending.

TKA and arthroscopy are procedures in which the instruments are routinely placed near the knee’s posterior capsule, and, therefore, very close to the vascular bundles. Orthopedic surgeons are well aware of this. While early kinds of lesions (thrombosis, hemorrhaging) are easier to detect sooner, the more delayed kinds (pseudoaneurysms and arteriovenous fistulas) also need to be considered for their early screening.

It is not possible to recommend systematic follow-up for this rare complication. However, given the high volume of this surgery (total knee arthroplasty), it is advisable to request bloodless studies (eco-Doppler) for monitoring in cases of clinical suspicion of this or other vascular complications (e.g., technical complexity, re-interventions, etc.).

## 4. Conclusions

This case uniquely highlights the successful long-term resolution of a rare and serious vascular complication following orthopedic knee surgery using endovascular treatment alone.

## Data Availability

The data presented in this study are available on request from the corresponding author due to privacy.
